# Intravenous immunoglobulin for the treatment of morphea in pediatric patients: A case series

**DOI:** 10.1016/j.jdcr.2025.05.049

**Published:** 2025-07-12

**Authors:** Milica Milakovic, Ronald M. Laxer, Elena Pope, Cathryn Sibbald

**Affiliations:** a Division of Dermatology, Department of Medicine, University of Toronto, Toronto, Ontario, Canada; b Division of Rheumatology, Department of Paediatrics, University of Toronto, Toronto, Ontario, Canada; c The Hospital for Sick Children, University of Toronto, Toronto, Ontario, Canada; d Division of Dermatology, Department of Paediatrics, The Hospital for Sick Children, University of Toronto, Toronto, Ontario, Canada

**Keywords:** ivIG, morphea, pediatric, scleroderma, treatment

## Introduction

Morphea, or localized scleroderma, is an inflammatory skin condition affecting the dermis and subcutaneous tissue, with potential involvement of deeper structures.^1^

Untreated, active disease can cause irreversible atrophy, contractures, and disability. Aggressive treatment with systemic corticosteroids, in combination with methotrexate or mycophenolate mofetil, is recommended by the consensus guidelines for linear, generalized, or pansclerotic subtypes.^2^

However, these treatments are not always well tolerated by patients, and some patients do not respond to these treatment approaches. Other treatments reported include abatacept, tocilizumab, and Janus Kinase inhibitors, all of which have their own associated side effects.^3^ Intravenous immunoglobulin (ivIG) is used in certain autoimmune dermatologic conditions and has been explored as an alternative treatment in morphea. ivIG’s immunomodulatory effects occur through various mechanisms, including the blockade of Fc receptors, inhibition of complement-mediated damage, alteration of cytokine profiles, reduction of circulating antibodies via anti-idiotype antibodies, and neutralization of autoantibody-triggering toxins.^4^ Several case reports have demonstrated the benefit of ivIG in treating morphea, possibly because of its immunomodulatory effects and antifibrotic properties.^4^

The safety and efficacy of ivIG for pediatric morphea have not been well studied. The present case series examines the treatment outcomes in 3 pediatric patients with morphea treated with ivIG.

**Fig 1:**
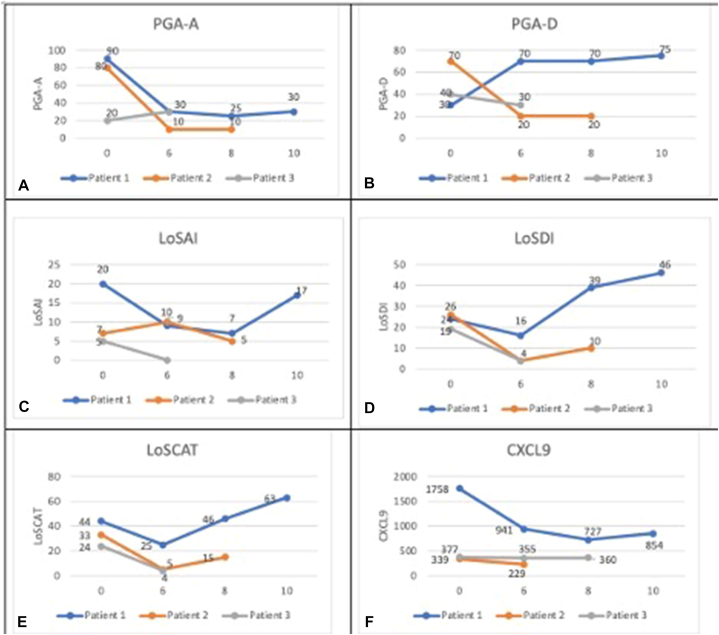
Progression of **(A)** PGA-A, **(B)** PGA-D, **(C)** LoSAI, **(D)** LoSDI, **(E)** LoSCAT, and **(F)** CXCL9 levels over the follow-up duration of the included cases.

All 3 patients were assessed and followed at the combined rheumatology-dermatology morphea clinic at SickKids. Assessments included the Physician Global Assessment of Activity (PGA-A) of morphea, Physician Global Assessment of Damage (PGA-D), Localized Scleroderma Cutaneous Assessment Tool (LoSCAT) score, assessing both activity and damage in localized scleroderma (Localized Scleroderma Skin Activity Index [LoSAI] and Localized Scleroderma Damage Index [LoSDI]), as well as CXCL9, a serum chemokine involved in immune response and inflammation identified as being a significant marker in morphea pathogenesis.^5^ Graphs demonstrating these outcomes are displayed in Fig 1.

## Case 1

Patient 1 is a 7-year-old girl who was diagnosed with morphea, mixed subtype (linear, deep circumscribed) at 5 years of age. At the time of diagnosis, she presented with hypopigmented macules on the left leg, with pruritus and pain. An magnetic resonance imaging (MRI) scan revealed edema of the deep fascia in the lower legs bilaterally, with left leg atrophy over the lateral knee. Assessment was also remarkable for arthritis of the left ankle and subtalar joint. She was negative for antinuclear antibodies and rheumatoid factor, and C-reactive protein was mildly elevated at 3.1 mg/L (ref range: 0.1-1 mg/L). No skin biopsy was performed.

Her initial treatment included 6 treatments of intravenous pulse corticosteroids (3 consecutive days monthly for 3 consecutive months, repeated once), and weekly subcutaneous methotrexate (15 mg/m^2^). Mycophenolate mofetil (MMF) (600 mg/m^2^/dose twice daily) was added due to persistent active disease after 4 months, in addition to 2 months of prednisone orally (2 mg/kg/day tapered slowly). Despite these interventions, her disease remained active with new areas of involvement after 2 years. As a result, in September 2023, she began monthly ivIG infusions at a dose of 1.69 g/kg/dose for 6 rounds. This dose was selected based on a review where pediatric patients were treated with doses of 1.5 to 2 g/kg, demonstrating efficacy. The slightly higher dose was chosen to account for individual disease severity and response.^4^

She developed aseptic meningitis after her first infusion, but subsequent infusions were well tolerated with the addition of a normal saline bolus, acetaminophen premedication, hydrocortisone, and rupatadine 30 minutes before infusion, as well as switching to a different brand of ivIG.

**Fig 2:**
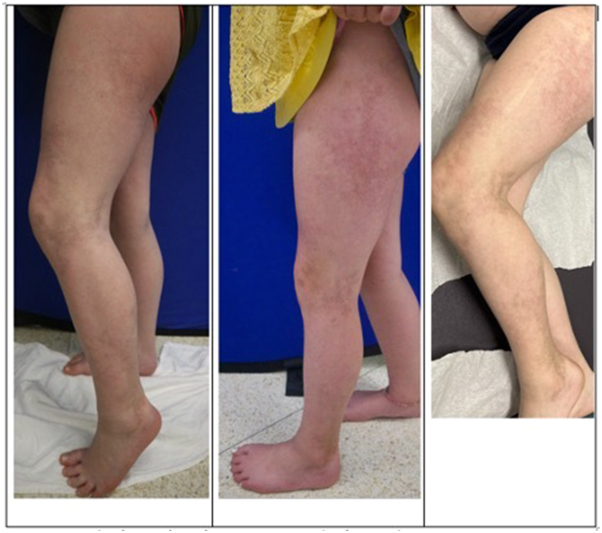
Clinical photos for patient 1. Baseline (preivIG), after ivIG ×6, and then 3 months after completing ivIG.

Throughout ivIG treatment period, PGA-A and LoSCAT (LoSAI and LoSDI) decreased, indicating improvement in disease. CXCL9 also decreased from 1,758 pg/mL to 941 pg/mL after completing 6 infusions and further to 727 at 8 months while on mycophenolate alone, with a normal reference for our laboratory of <657 pg/mL. Unfortunately, this benefit was not maintained. Four months after stopping ivIG, she developed new lesions on the ankles with an increase in her LoSAI and CXCL9, and ivIG at 1 g/kg/dose has been restarted. She received only 1 full dose, and the second dose was cut short because of an urticarial eruption and is now on tofacitinib. Given the reactions observed in patient 1, we elected to use 1 g/kg dosing both upon restarting treatment with her and with the other 2 patients. Photographs are included in Fig 2.

## Case 2

Patient 2 is a 10-year-old girl diagnosed at 9 years of age with linear morphea, affecting the upper limbs. She initially presented with a xerotic patch on the right shoulder that expanded and deepened, with the development of sensitivity on palpation, but without pruritus. A skin biopsy performed at another clinic was interpreted as granuloma annulare. However, a second biopsy performed a year later demonstrated histopathologic skin findings consistent with morphea. She did not develop arthritis or other extracutaneous manifestations at the time of diagnosis or during follow-up.

An MRI scan revealed an indentation of soft tissues of the right upper extremity with a vague signal abnormality. Serum antinuclear antibodies and antidouble-stranded DNA were positive (1:640 and 34.4, respectively), but no stigmata of systemic lupus erythematosus. Initial treatment included 3 months of pulse intravenous steroids and MMF (600 mg/m^2^/dose twice daily). Despite this, her disease activity persisted, with ongoing symptoms in the right arm, persistent waxy skin induration, and elevated C-reactive protein levels. Consequently, ivIG therapy was initiated at 1 g/kg/dose per month for 6 months. She completed 6 planned rounds of ivIG in conjunction with continued MMF and topical calcipotriol/betamethasone dipropionate. She remains in remission at the 6-month follow-up.

**Fig 3:**
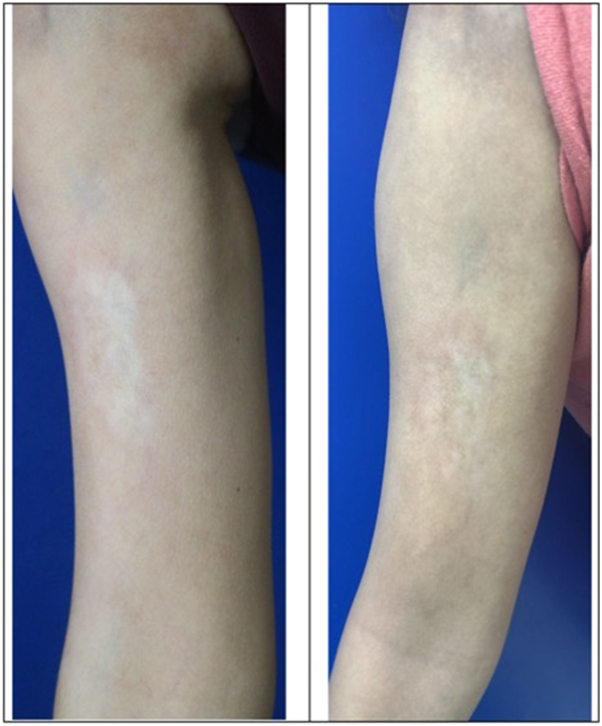
Clinical photos for patient 2. Baseline (preivIG) and then after ivIG ×6.

The patient reported mild nausea after the first 2 doses of ivIG, but there was clear improvement in her skin, with no progression of disease after 3 doses of ivIG. Her PGA-A, PGA-D, LoSCAT, LoSAI, and LoSDI and CXCL9 all decreased after 6 months of ivIG and stayed relatively stable 2 months after stopping (Figs 1 and 3).

## Case 3

Patient 3 is a 13-year-old girl diagnosed with linear morphea affecting the left arm at the age of 10 years. At diagnosis, she reported no pruritus or pain, and her medical history was unremarkable. Extracutaneous involvement included tenosynovitis, myositis, fasciitis, and osseous involvement, evident on an MRI scan. No skin biopsy was performed, and baseline blood tests were within normal limits.

Initial treatments included MMF (600 mg/m^2^/dose twice daily), 2 rounds of intravenous methylprednisolone (1 g intravenously for 3 consecutive days for 3 consecutive months, repeated once), and topical calcipotriol/betamethasone dipropionate. Because of significant gastrointestinal side effects and poor compliance with MMF, subcutaneous methotrexate (15 mg/m^2^) weekly was started after 3 months, and MMF was stopped. A transient increase in liver enzymes was observed that resolved without intervention. She acknowledged poor adherence to methotrexate (missing 1 or 2 doses monthly), along with nausea and vomiting while on the drug. An MRI scan revealed interval improvement of osseous involvement within the left arm after 20 months of treatment, but the cutaneous plaque remained infiltrated with persistent erythema at the border.

**Fig 4:**
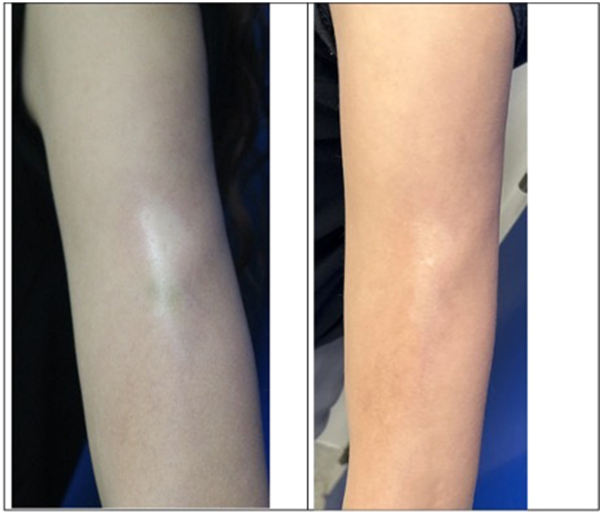
Clinical photos for patient 3. Baseline (preivIG) and then after ivIG ×6.

She was started on ivIG at a dose of 1 g/kg for 6 total doses. She reported headache and diarrhea after the first ivIG infusion and developed impetigo on the face, which cleared with topical mupirocin (felt to be coincidental). Blood work monitoring during ivIG therapy remained within normal limits. After 6 rounds of ivIG, PGA-A increased from 20 to 30, whereas PGA-D, LoSCAT, LoSDI, LoSAI scores, and CXCL9 decreased (Figs 1 and 4). She remains in remission at the 6-month follow-up.

## Discussion

We report 3 female pediatric patients with linear and mixed subtypes of morphea treated with ivIG due to inadequate responses to standard treatment. All 3 patients demonstrated improvement in disease activity measures over the course of their ivIG treatment, and with the exception of 1 episode of aseptic meningitis and 1 patient with gastrointestinal upset, the ivIG was well tolerated by all.

These data contribute to the expanding body of evidence for ivIG in morphea, specifically for refractory disease. An adult case series of 3 patients with recalcitrant generalized morphea treated with the addition of ivIG reported improved skin activity and joint mobility with minimal adverse effects.^4^

Only a small number of reports involve children. In 1 case report, an 11-year-old boy with pansclerotic morphea treated with ivIG 1.2 g/kg over 5 days monthly had considerable improvement in disease without adverse effects.^6^ However, 2 cases of children with morphea and eosinophilic fasciitis treated with ivIG and other immunosuppressive medication reported only slow or partial response.^4^

Our findings align with those of previous reports demonstrating ivIG efficacy in refractory morphea. A case of a 35-year-old patient with generalized morphea, unresponsive to methotrexate and mycophenolate mofetil, showed clinical improvement with high-dose ivIG and methotrexate, including reduced skin induration and improved joint mobility, without significant adverse effects. A systematic review of 42 cases further reported 85.7% achieving clinically relevant responses, with ivIG combined with immunosuppressants like MMF or methotrexate yielding the best outcomes. These findings support ivIG as a promising third-line therapy for severe, refractory morphea due to its immunomodulatory effects.^7^

In this case series, changes in CXCL9 were measured pre- and post-ivIG treatments. CXCL9 has been identified as a key biomarker correlated with disease activity in patients with active morphea. IvIG downregulates proinflammatory cytokines, promotes Tregs, and interferes with Fc receptor-activation, leading to reduced inflammation and lower levels of CXCL9, a downstream product of IFN-γ primarily produced by cutaneous macrophages.^5^^,^^8^ From our case series, CXCL9 decreased after 6 rounds of ivIG, which aligns with its expected role as a marker of inflammation. However, the absence of a control group limits the ability to attribute these changes definitively to ivIG therapy.

Of note, in the patient with longer follow-up post-ivIG infusions, there were signs of relapse after 4 months of stopping the ivIG. This suggests that the benefit of ivIG may not be maintained, and more data are needed to better inform the optimal duration of this modality treatment and any patient or disease characteristics that may influence this.

Our data highlight the potential efficacy of ivIG as a treatment option for pediatric patients with morphea, particularly in cases where standard treatments have failed or are poorly tolerated. In addition to improving clinical disease, the long-term safety of ivIG in pediatrics offers a benefit compared with other third-line treatments such as abatacept, tocilizumab, or Janus Kinase inhibitors.

## Conclusion

In conclusion, ivIG, in combination with steroid-sparing agents, appears to be an effective and safe adjunctive treatment option for patients with refractory morphea. Future research should focus on larger, controlled, or randomized studies with long-term follow-ups to further elucidate the safety and efficacy of ivIG in the pediatric population. In addition, further exploration of biomarkers such as CXCL9, as well as increased reporting and measuring of relevant biomarkers in the published literature, could provide more insight into disease activity and therapeutic responses in morphea.

## Conflicts of interest

None disclosed.
